# Effectiveness of revascularization interventions compared with medical therapy in patients with ischemic cardiomyopathy: A systematic review protocol

**DOI:** 10.1097/MD.0000000000009958

**Published:** 2018-03-09

**Authors:** Aziz Rezapour, Saeed Bagheri Faradonbeh, Vahid Alipour, Mani Yusefvand

**Affiliations:** a Health Management and Economics Research Center, Iran University of Medical Sciences, Tehran, Iran; b Department of Health Economics, School of Health Management and Information Sciences, Iran University of Medical Sciences, Tehran, Iran; c Department of Health Management and Economics, School of Public Health, Tehran University of Medical Sciences, Tehran, Iran.

**Keywords:** effectiveness, medical therapy, revascularization interventions, systematic review protocol

## Abstract

**Background:**

Cardiomyopathies is a group of heart diseases that directly affects the heart muscle, and their causes is not just high blood pressure, congenital and pericardial diseases but ischemic cardiomyopathy disease are also caused by vascular disorders, and to confirm the diagnosis, angiography is required. There are several methods for treating and controlling ischemic cardiomyopathy in world health systems and especially in the Iran health system, which include medical treatment, percutaneous coronary intervention (PCI), and coronary artery bypass graft (CABG).

**Methods:**

This systematic review will includes observational and interventional studies in English and Persian languages and evaluates effectiveness of revascularization interventions and medical therapy in patients with ischemic cardiomyopathy. Animal studies will not be considered. In this systematic review, our sources of information will be electronic databases, trial registries, and different types of grey literature. An electronic search is performed through PubMed, Cochrane library, Scopus, Web of Science, EMBASE, Tufts Medical Center Cost-Effectiveness Analysis Registry, NHS Economic Evaluations Database. To integrate the results of studies with similar results, meta-analysis will be used, for which Comprehensive Meta-Analysis (CMA) software will be used. Results are provided using relative risk with a 95% confidence interval for information.

**Results:**

The results of this systematic review will be published in a peer-reviewed journal.

**Conclusion:**

To our knowledge, this systematic review will be the first to evaluate existing research on the effectiveness of revascularization interventions compared with medical therapy in patients with ischemic cardiomyopathy. The review will benefit patients, healthcare providers, and policymakers.

## Abstract 2

Supplemental Digital Content is available in the text

**Key Points**

- This systematic review will develops a consensus on the effectiveness of revascularization interventions compared with medical therapy in medical articles.
- In this systematic review, databases in languages other than English and Persian (French, German, Chinese, etc) will not be searched or included. This limitation may cause language bias.

## 1 Introduction

Heart ischemic diseases, a group of heart failure, are categorized into 2 acute and chronic groups, and its chronic category is cardiovascular atherosclerotic, aneurysm, ischemic cardiomyopathy, and myocardial ischemia. Cardiac ischemic disease is a condition that reduces blood flow to the heart muscle, which can disrupt the flow of blood through coronary arteries, most often it happens because of atherosclerotic stenosis, but sometimes also because of arterial spasm.^[1,2]^ Cardiomyopathy is also a group of heart diseases that directly affects the heart muscle and is not only due to high blood pressure, congenital, pericardial disease, but also cardiomyopathy ischemia is due to vascular disorders, and to confirm this diagnosis, angiography is required.^[3]^

There are several methods for treating and controlling ischemic cardiomyopathy in world health systems and especially in the Iran health system, which include:

- Medical therapy, such as the use of anticoagulants such as thrombolytic, beta blocker, calcium blocker, antiarrhythmic, nitrates, diuretics, antiplatelet, and lipid regulating drugs.^[4,5]^
- Percutaneous Coronary Intervention, including piercing the skin to access the femoral artery by catheter, guide catheter-guided balloon to narrow or blocked coronary artery, inflating the balloon and dilating coronary stenting to prevent reblockage.^[2,6]^
- Coronary Artery Bypass Surgery includes the creation of bypass with saphenous vein or arterial graft pieces of breast through open narrowed or blocked coronary sternum in place.^[6]^ This is a surgical technique that involves opening the chest and the tight and closed coronary artery and is usually done by using the vein or artery from other parts of the body. The advantages of this surgical technique include angina relief in 60% to 90% patients in the first year, a significant reduction in the mortality of the disease when combined with drug therapy, and a reduction in revascularization after 1 year, and its disadvantages are high costs, in particular hospital care costs, and an increase in myocardial infarction rate compared with drug therapy.^[7,8]^

It is necessary to explain that coronary artery bypass graft (CABG) and percutaneous coronary intervention (PCI) are part of the ischemic myocardial revascularization techniques that are used to relieve coronary artery obstruction and therefore their use in patients with ischemic cardiomyopathy relieves pain and heals the patient.^[9]^

So far, no systematic reviews have been made on this topic, But here are the results of some relevant studies. Medical treatment for coronary disease has advanced dramatically in recent years and produced prognostic benefits in the context of properly designed, randomized, controlled trials. Surgical techniques have also advanced but it is difficult to be sure that they have really reduced mortality because such comparisons are retrospective rather than concurrent. Even if the proportionate benefits from surgery were to increase, the falling mortality with optimal medical therapy will reduce the absolute benefits of surgery over medical treatment. Thus, the surgical interventions are being superseded and their relevance to modern medical practice must be questioned,^[5,10]^ and in a clinical trial study in the field of drug therapy compared with surgery in patients with ischemic heart disease, there was no significant evidence of the benefit of these 2 therapies in reducing mortality and morbidity^[11]^; in another study, the effect of surgical intervention was similar to that of drug therapy in patients with heart disease,^[12]^ and in a meta-analysis study, surgical intervention, compared with drug therapy, led to greater reduction in the mortality rate of patients with heart disease.^[13]^

Carrying out systematic review studies and determining the effectiveness of cardiovascular interventions plays an important role in informing about reimbursement decisions, health care pricing, providing clinical guidance on the use of existing clinical technologies, interventions strategic purchasing, targeted health care provision, and the production of scientific evidence for policy decisions and ultimately, the optimal allocation of financial resources for health in the field of cardiovascular disease.^[14–16]^

This study is performed with the following question in mind:

- Which treatment interventions are more effective in treating patients with ischemic cardiomyopathy?

The objectives of this study are:

Primary objective:

- Effectiveness of revascularization interventions compared with medical therapy in patients with ischemic cardiomyopathy

Secondary objectives:

- Determining the effectiveness of treatment by using revascularization interventions in patients with ischemic cardiomyopathy
- Determining the effectiveness of treatment by using drug therapy in patients with ischemic cardiomyopathy

## 2 Methods and analysis

### 2.1 Eligibility criteria

#### 2.1.1 Study characteristics

This systematic review includes observational (case report, case series, cross-sectional, case–control, cohort, etc) and interventional (quasi-experimental studies, randomized controlled trials, community trials, field trials, etc) studies in English and Persian language and examines the effectiveness of revascularization and medical therapy interventions in patients with ischemic cardiomyopathy. Animal studies are not considered. The Preferred Reporting Items for Systematic reviews and Meta-Analyses for Protocols 2015 (PRISMA-P 2015) and SPIRIT guidelines have been used for preparing and reporting the protocol of this systematic review.^[17]^

#### 2.1.2 Types of participants

This systematic review targets studies in patients with heart failure with ejection fraction < 35% who have angiography, surgery, or medical treatment.

#### 2.1.3 Setting and time frame

In this systematic review, all theses, reports, and relevant studies during 1980 to 2017 are considered.

#### 2.1.4 Report characteristics

Only articles that have abstract in English and studies whose full text is available are chosen. No limitation is considered for date of acceptance or publication. As for publication status, we consider only articles that are published or in press.

#### 2.1.5 Information sources

Our sources of information include electronic databases, trial registries, and different types of grey literature. An electronic search is performed through PubMed, Cochrane library, Scopus, Web of Science, EMBASE, Tufts Medical Center Cost-Effectiveness Analysis Registry, NHS Economic Evaluations Database. To identify appropriate key words, in addition to MESH terms, popular and commonly-used phrases stated in the related literature is utilized. First, the search strategy is developed and completed in PubMed, and then the same strategy is applied to other databases. Other sources are searched to identify related grey literature. ProQuest is searched for dissertations. Meeting abstracts are searched through SCOPUS, web of science, and pertinent websites. Reference lists of relevant articles and systematic reviews, and tables of contents of key journals in this field are searched as well.

### 2.2 Search strategy

Our initial search syntax for PubMed will be:

1. “effectiveness”[Title/Abstract] OR “efficacy”[Title/Abstract]) OR “evaluation”[Title/Abstract]
2. “medical therapy”[Title/Abstract] OR “drug therapy”[Title/Abstract]) OR “medical treatment”[Title/Abstract]
3. “thrombolitic”[Title/Abstract] OR“betablocker”[Title/Abstract] OR“calcium blocker”[Title/Abstract] OR“antiarrhytmic”[Title/Abstract] OR“nitrates”[Title/Abstract] OR“diuretic”[Title/Abstract] OR“antiplatelet”[Title/Abstract] OR“lipid regulating”[Title/Abstract]
4. “Revascularization”[Title/Abstract] OR “Percutaneous Coronary Intervention”[Title/Abstract]) OR “ Coronary Artery Bypass Graft ”[Title/Abstract]
5. “Percutaneous Coronary Intervention”[Title/Abstract] OR“PCI”[Title/Abstract] OR “Percutaneous Coronary Revascularization”[Title/Abstract] OR“angioplasty”[Title/Abstract] OR“baloon”[Title/Abstract] OR“Cardiovascular Surgical Procedure”[Title/Abstract] OR“Atherectomy”[Title/Abstract]
6. “Coronary Artery Bypass Graft”[Title/Abstract] OR “ Coronary Artery Bypass Surgery”[Title/Abstract]) OR “CABG”[Title/Abstract] OR “surgical procedure”[Title/Abstract] OR “vascular grafting”[Title/Abstract]
7. “Ischemic”[Title/Abstract] OR “Ischemia”[Title/Abstract]) OR “Myocardial Ischemia ”[Title/Abstract]
8. “cardiomyopathy”[Title/Abstract]
9. 1 AND 2 AND 7 AND 8
10. 1 AND 4 AND 7 AND 8
11. 1 AND 2 AND 5 AND 6 AND 7 AND 8
12. 1 AND 3 AND 5 AND 6 AND 7 AND 8

### 2.3 Study records

#### 2.3.1 Selection process

Two authors independently perform the primary article screening. First they review the title and abstract of the articles independently and then their selected articles will be categorized into 2 groups: relevant and irrelevant. Articles categorized as irrelevant by both reviewers are eliminated from the study. Then each reviewer reviews the full text of the remaining articles and makes a list of articles to be included. The 2 lists are then compared and nonconformities will be discussed. When an agreement is not reached, the whole team will make the final decision.

#### 2.3.2 Data management

Data are extracted from papers and entered into data sheets independently by 2 reviewers. These 2 sheets and their differences are checked by a third reviewer. Any potential difference among reviewers is discussed within the team and if not resolved, the manuscript authors will be contacted.

### 2.4 Data items

From each article, the following information are extracted: article ID, author, publication year, study design, sample size, and the aim of the study and number of deaths and admissions.

### 2.5 Data collection

A data extraction form is developed (Appendix 1), and study data are independently assessed and extracted by 2 reviewers

### 2.6 Data synthesis

After searching for studies, the quality of all studies is evaluated by the Jadad score (Appendix 2), which had given a score between 0 and 5 based on criteria such as randomization, blindness, and a decrease in the number of samples during the study. Score ≥3 in terms of acceptable quality and score < 3 are considered as an exclusion criterion.^[18]^ To integrate the results of studies with similar results, meta-analysis is used, for which Comprehensive Meta-Analysis (CMA) software is used. Results are provided using relative risk with a 95% confidence interval for information. *P* < 0.05 was statistically significant.^[19]^ To test heterogeneity, the *I*^2^ test is used and if there is a heterogeneity or lack of studies, the random effects method is used. Funnel chart is used as an indicator of publication bias. To illustrate meta-analysis results, an accumulation chart is used. This chart is the most common type of diagram in meta-analysis that displays the information of each individual study and its final outcome.^[18]^

## 3 Discussion

This protocol presents the methodology of a systematic review for evaluating effectiveness of revascularization interventions compared with medical therapy in patients with ischemic cardiomyopathy.

So far most of the studies that have been done are in relation to comparison of effectiveness of CABG versus PCI, effect of PCI on Survival, effectiveness of cardiac resynchronization therapy by the frequency of revascularization procedures in ischemic cardiomyopathy patients,^[20–22]^ or have studied one of the effectiveness indicators in their studies.^[23,24]^ To our knowledge, this systematic review will be the first to evaluate existing research on the effectiveness of revascularization interventions compared with medical therapy in patients with ischemic cardiomyopathy. The review will benefit patients, healthcare providers, and policymakers.

## Supplementary Material

Supplemental Digital Content
