# Supplementary material for: Effectiveness of revascularization interventions compared with medical therapy in patients with ischemic cardiomyopathy: A systematic review protocol
Source: Medicine (Baltimore). 2018 Mar 9;97(10):e9958. doi: 10.1097/MD.0000000000009958 (PMC5882430; doi:10.1097/MD.0000000000009958)
Supplement: Supplemental Digital Content [file medi-97-e9958-s001.docx]

Appendix 1 : Study Data Extraction Form

| Article  ID | author | publication year | study design | sample size | The aim of the study | Number of deaths and admissions |
| --- | --- | --- | --- | --- | --- | --- |
|  |  |  |  |  |  |  |

Appendix 2: assessment of studies quality with using Jadad score

| Study | Randomization | Double-blind | Expression of the number of samples during the study | Overall rating |
| --- | --- | --- | --- | --- |
|  |  |  |  |  |
